# Developing a customised approach for strengthening tuberculosis laboratory quality management systems toward accreditation

**DOI:** 10.4102/ajlm.v6i2.576

**Published:** 2017-03-31

**Authors:** Heidi Albert, Andre Trollip, Donatelle Erni, Kekeletso Kao

**Affiliations:** 1 Foundation for Innovative New Diagnostics (FIND), Cape Town, South Africa; 2 Foundation for Innovative New Diagnostics (FIND), Geneva, Switzerland

## Abstract

**Background:**

Quality-assured tuberculosis laboratory services are critical to achieve global and national goals for tuberculosis prevention and care. Implementation of a quality management system (QMS) in laboratories leads to improved quality of diagnostic tests and better patient care. The Strengthening Laboratory Management Toward Accreditation (SLMTA) programme has led to measurable improvements in the QMS of clinical laboratories. However, progress in tuberculosis laboratories has been slower, which may be attributed to the need for a structured tuberculosis-specific approach to implementing QMS. We describe the development and early implementation of the Strengthening Tuberculosis Laboratory Management Toward Accreditation (TB SLMTA) programme.

**Development:**

The TB SLMTA curriculum was developed by customizing the SLMTA curriculum to include specific tools, job aids and supplementary materials specific to the tuberculosis laboratory. The TB SLMTA Harmonized Checklist was developed from the World Health Organisation Regional Office for Africa Stepwise Laboratory Quality Improvement Process Towards Accreditation checklist, and incorporated tuberculosis-specific requirements from the Global Laboratory Initiative Stepwise Process Towards Tuberculosis Laboratory Accreditation online tool.

**Implementation:**

Four regional training-of-trainers workshops have been conducted since 2013. The TB SLMTA programme has been rolled out in 37 tuberculosis laboratories in 10 countries using the Workshop approach in 32 laboratories in five countries and the Facility-based approach in five tuberculosis laboratories in five countries.

**Conclusion:**

Lessons learnt from early implementation of TB SLMTA suggest that a structured training and mentoring programme can build a foundation towards further quality improvement in tuberculosis laboratories. Structured mentoring, and institutionalisation of QMS into country programmes, is needed to support tuberculosis laboratories to achieve accreditation.

## Introduction

The World Health Organization’s (WHO) End TB Strategy calls for an end to the global tuberculosis epidemic. It aims to reduce deaths by 95% and new tuberculosis cases by 90% and to ensure that no family is burdened with catastrophic expenses due to tuberculosis by 2025.^1^ Despite the fall in global tuberculosis mortality by 47% since 1990, the disease still claimed more than 1.5 million lives in 2014.^2^ A cascade of events, including poor screening, failure to link screened patients to diagnostic services, and failure to link diagnosed patients to treatment, means that many people die from tuberculosis due to delayed diagnosis and treatment initiation.^3^

Quality-assured laboratory services are critical for the provision of timely, accurate and reliable results to support diagnosis, drug-resistance testing, treatment monitoring and surveillance of disease. Weak laboratory systems result in high levels of laboratory error that impact patient care and undermine the confidence healthcare providers have in laboratory services.^4^ In recent years, the focus on improving laboratory quality management systems (QMS), and assuring the quality of laboratory services by working toward national or international laboratory accreditation has intensified.^5^ Accreditation is the formal recognition of implementation of a QMS that adheres to international standards and has been shown to improve the quality of healthcare for patients through reduction in testing errors.^6^

The Strengthening Laboratory Management Toward Accreditation (SLMTA) programme was developed by the United States Centers for Disease Control and Prevention in collaboration with the American Society for Clinical Pathology, the Clinton Health Access Initiative, and the WHO Regional Office for Africa to promote immediate and measurable quality improvement in laboratories in developing countries. SLMTA is a programme that may be used to prepare laboratories for accreditation.^7^ Since its launch in Kigali, Rwanda in 2009, SLMTA has been implemented in 47 countries (23 in Africa), with 617 laboratories already enrolled. Eighteen per cent of the enrolled laboratories are at the national level and most (98%) are providing HIV-related services.^8^ Only four National Tuberculosis Reference Laboratories (NTRLs) in Africa have achieved international accreditation to date,^9,10^ and only six NTRLs have undergone a formal Stepwise Laboratory Quality Improvement Process Towards Accreditation (SLIPTA) audit by the African Society for Laboratory Medicine (T. Mekonen, personal communication). Accredited NTRLs are better equipped to support the national tuberculosis laboratory network and also provide reliable support to their national tuberculosis control and treatment programmes.^11^

Since 2007, the Foundation for Innovative New Diagnostics (FIND) has worked with Ministries of Health to introduce new diagnostic technologies to improve the diagnosis of tuberculosis, detection of drug resistance^12^ and upgrading of facilities.^13,14,15,16^ Although technical capacity to conduct new tests can be developed within a relatively short time frame, persistent challenges to providing quality results in a consistent manner often remain, many of which are linked to laboratory quality system weaknesses. In 2011, through funding from the United States President’s Emergency Plan for AIDS Relief, FIND was involved in implementation of the SLMTA programme in clinical laboratories in Dominican Republic. Measurable improvement was observed in cohorts of laboratories participating in the programme. However, tuberculosis laboratories were not included in this programme. Concurrently, the Global Laboratory Initiative (GLI) was developing its Stepwise Process Towards Tuberculosis Laboratory Accreditation online tool.^17^ This tool provided online resources and a framework consisting of four phases, but did not have training materials or an implementation plan to enable adoption by tuberculosis laboratories. Tuberculosis laboratories, particularly at the central or regional-level, have separate facilities from other clinical laboratories. They have different requirements for biosafety and quality assurance, and have often been excluded from accreditation efforts. Recognising the unique needs of tuberculosis laboratories, FIND developed a comprehensive approach to tuberculosis laboratory strengthening based on the existing SLMTA approach and incorporating aspects of the GLI Stepwise Process Towards Tuberculosis Laboratory Accreditation online tool.

In this article, we describe the development of the Tuberculosis Strengthening Laboratory Management Toward Accreditation (TB SLMTA) programme and challenges experienced during early implementation in 10 countries. We also reflect on approaches that will ensure continued quality improvement to reach accreditation and institutionalisation of the programme.

## TB SLMTA development

### Customisation of training materials

In 2012, FIND conducted a review of the SLMTA materials, and customised the content for tuberculosis laboratories based on available tuberculosis resources (either developed internally by FIND or by other organisations). This customisation included the development of specific tools, job aids and supplementary materials for the implementation of a QMS in the tuberculosis laboratory (Table 1), but kept the overall structure of the SLMTA curriculum. Customisation included major changes to the content of the SLMTA *Facilities and Safety* and *Quality Assurance* modules (the focus was changed from the quantitative testing in SLMTA to the qualitative and semi-quantitative testing relevant to the tuberculosis laboratory). The SLMTA *Laboratory Testing* and *Test Result Reporting* modules were combined and an *Auditing* module was introduced. Tuberculosis laboratory-specific tools, examples and scenarios were introduced throughout all modules in the training. The TB SLMTA Harmonized Checklist was also introduced as part of the programme.

The TB SLMTA curriculum was piloted in Cape Town in April 2013 in a shortened Training-of-trainers (TOT) Workshop led by SLMTA Master Trainers and with experienced tuberculosis laboratory specialists as participants. Following the pilot workshop, some changes were made to the training materials (e.g. organisation and cross-referencing of tools, adjustment of training notes for clarity, and editing errors) and the TB SLMTA Harmonised Checklist was revised.

Subsequent review and revision of the TB SLMTA curriculum has been conducted to keep the content current with an updated GLI tool (version 2.0, 2013) and WHO Regional Office for Africa SLIPTA (2015) tool. A review of the TB SLMTA curriculum was conducted in 2015 due to experience that improvement projects did not necessarily target the highest priority non-conformities. Based on feedback from previous trainings, minor changes were also made to the *Cross-cutting*, *Facilities and Safety* and *Quality Assurance* modules.

### TB SLMTA Harmonized Checklist

The TB SLMTA Harmonized Checklist^18^ is based on the WHO Regional Office for Africa SLIPTA checklist (2007),^19^ and incorporates tuberculosis laboratory-specific requirements as provided in the GLI Stepwise Process Towards Tuberculosis Laboratory Accreditation tool, which were inserted as sub-clauses in the SLIPTA checklist. The TB SLMTA Harmonized Checklist is used to assess the QMS of the tuberculosis laboratory prior to enrolment in the programme (baseline assessment) and after programme completion (exit assessment). The differences between the scores obtained overall and for each section, are a measure of the impact of the programme. Assessors evaluate the laboratory operations as per checklist items, scoring the assessment and documenting their findings in detail.

**TABLE 1:** Comparison of SLMTA and TB SLMTA programme components.

Component	SLMTA	TB SLMTA
Training-of trainer workshop	Yes	Yes
Implementation models	2 (workshop and facility-based)	2 (workshop and facility-based)
Checklist	WHO AFRO SLIPTA Checklist v1: Developed: 2009 Based on: ISO 15189 (2007) Total points: 258 WHO AFRO SLIPTA Checklist v2: Developed: 2015 Based on: ISO 15189 (2012) Total points: 275	TB SLMTA Harmonized Checklist v1: Developed: 2012 Based on: ISO 15189 (2007) & GLI Total points: 258 TB SLMTA Harmonized Checklist v2.1: Developed: 2016 Based on: ISO 15189 (2012) & GLI Total points: 275
Modules	Introduction^20^ Cross Cutting Productivity Management Work Area Management Inventory Management Procurement Management Maintenance of Equipment Quality Assurance Specimen Collection and Processing Laboratory Testing Test Result Reporting Documents and Record Management	Introduction Cross-cutting Introduction to QMSProductivity management Safety Inventory management Procurement management Equipment management Quality Assurance Specimen management Result management & customer service Documents & record management Auditing
Mentoring	Yes (various- embedded mentor model encouraged)^30^	Yes (short-term model)
Improvement projects	Yes (one simple, one complex)	Yes (two complex; specific focus on addressing pre-assessment areas of weaknesses)
Supplementary	None	Online Introduction to QMS course Online Biosafety course Pre-training English language assessment

SLMTA, Strengthening Laboratory Management Toward Accreditation; TB SLMTA, Strengthening Tuberculosis Laboratory Management Toward Accreditation; WHO AFRO SLIPTA, World Health Organisation Regional Office for Africa Stepwise Laboratory Quality Improvement Process Towards Accreditation; ISO, International Organization for Standardization; GLI, Global Laboratory Initiative; QMS, quality management systems.

The pilot version of the TB SLMTA Harmonized Checklist^20^ had additional scores allocated to the tuberculosis-specific clauses. A revised checklist (TB SLMTA Harmonized Checklist v1.0), which maintained the original SLIPTA scoring system,^21^ was used in the TB SLMTA roll-out. Recognition is given using a five-star grading system, with the following scores corresponding to the indicated number of stars: zero stars (0–142 points; < 55%), one star (143–165 points; 55–64%), two stars (166–191 points; 65%–74%), three stars (192–217 points; 75%–84%), four stars (218–243 points; 85%–94%) and five stars (244–258 points; ≥ 95%).

The TB SLMTA Harmonized Checklist 1.0 was recently revised in keeping with SLIPTA v2:2015, and the additional clauses of International Organization for Standardization 15189:2012. The questions added pertain to risk assessment, laboratory information system, contingency planning and safety. The TB SLMTA Harmonized Checklist v1.0 is available in English and Spanish. The TB SLMTA Harmonized Checklist v2.1 is available in English and Russian.^16^

### Implementation of TB SLMTA

**FIGURE 1:**
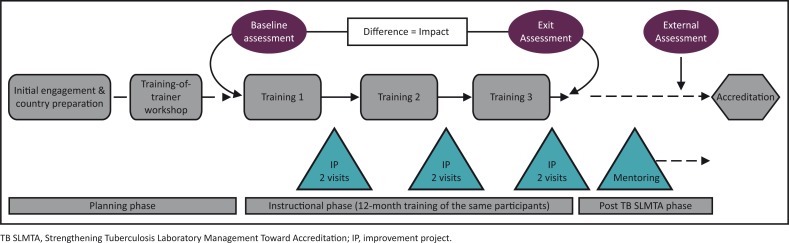
Diagrammatic representation of the TB SLMTA programme from initiation to accreditation

Implementation of the TB SLMTA programme starts with the initial engagement with the Ministry of Health on the programme scope and expected outputs, as well as commitments required from the country (Figure 1). During this planning phase, the country selects the participating tuberculosis laboratories, the model of implementation, the trainees to attend the TOT and the TB SLMTA participants who will attend the in-country training. Countries selects two or three participants per laboratory to attend the in-country TB SLMTA training. Typically, participants include the laboratory manager, quality officer and one technician. After graduation from the TOT, the certified trainers implement the programme in the country. Baseline and exit assessments are conducted with the TB SLMTA Harmonized Checklist v1.0 by trainers or SLIPTA-trained assessors with tuberculosis laboratory experience. In-country national or regional trainings are conducted over a period of 12–15 months. Between trainings, participants work on improvement projects supervised by the TB SLMTA mentors. Post-TB SLMTA activities are conducted in the laboratories under supervision of the mentors before an external assessment determines the readiness for accreditation.

### Training-of-trainers workshop

The TB SLMTA TOTs are conducted by SLMTA Master Trainers, and are based on teach-back methodology.^22^ This practice-based training approach requires trainees to play the roles of both trainer and participant as they teach the curriculum at the same time as they are learning the content. The TOTs provide trainees with an introduction to the TB SLMTA materials, practice in delivering the content and receiving feedback on their performance. The ratio of trainees to Master Trainers is a maximum of eight to one. To certify as trainers, trainees must demonstrate knowledge of TB SLMTA curriculum and proficiency in delivering training. Trainees that find teach-back challenging and do not show a good understanding of the materials graduate as one-one coaches. They can facilitate rollout in their laboratory, but are not certified to train others.

Mentors are trainers who support the in-country training participants during the implementation phases between trainings. During mentoring visits to the laboratory, they supervise the participants as they implement the improvement projects, and provide resources (e.g. Standard Operating Procedures) to implement what was taught in the training in the tuberculosis laboratory. The fundamentals of mentoring are modelled during the TOT. Trainees, who are certified as trainers, and who show an aptitude for mentoring are selected by the Master Trainers to perform mentoring in their countries. Mentoring in TB SLMTA builds on the relationship established between trainer and participant, and seeks to support programme implementation in the laboratory. Master trainers support the certified trainers and mentors during their first national or regional training, and where possible provide at least one interim visit to support mentoring. Trainers under supervision receive additional support from the Master Trainers during the workshop and, if assessed as proficient, can then graduate as trainers.

The TOTs are intensive and highly interactive hence good language skills and a working knowledge of QMS concepts is required. Based on this observation and challenges experienced in conducting a TOT with participants with varying levels of English fluency, a mandatory online training was introduced prior to the TOT, based on the WHO *Laboratory Quality Management System: Handbook*,^23^ to ensure that trainees have a basic understanding of QMS principles. In addition, trainees whose first language is not English are required to successfully complete an online language competency training before registration for the TOT.

### Models for implementation

Two models have been adopted for implementation of TB SLMTA:

1. *Workshop approach*: Where several tuberculosis laboratories are available in-country (or in cases where more than one country conducts centralised trainings), the three-workshop approach can be used. Three five-day regional workshops are conducted by trainers, approximately three months apart.
2. *Facility-based approach*: Where there is only one tuberculosis laboratory in the country being enrolled in TB SLMTA the facility-based approach may be used. The facility-based approach follows the same TB SLMTA curriculum, with training sessions split into three blocks over 12 months.

Factors affecting choice of implementation model include funding, number of laboratories participating in TB SLMTA, and availability of staff. TB SLMTA is targeted for implementation in tuberculosis laboratories at the national or referral level. These laboratories are conducting advanced tuberculosis testing, and generally have separate facilities from general laboratories. Laboratories conducting tuberculosis testing on lower levels of the healthcare system are not targeted with this training. However, this does not preclude the use of TB SLMTA resources to guide them, especially those related to safety and quality assurance.

### Improvement projects and mentoring

Improvement projects are broad-based activities that address weaknesses in the QMS. Topics for improvement projects are chosen from subjects covered in the trainings. As with SLMTA (Table 1), each participant is required to complete two improvement projects between trainings. The ‘just do it’ project (e.g. maintaining personnel files) is implemented as a group by all the participants from the laboratory. The ‘complex’ project, which requires extensive planning and before-and-after data collection, is chosen with assistance from the certified trainers. Ideally, the laboratory management is included in the decision of the topic and scope (if laboratory managers are not participants), to ensure management engagement and allocation of time and resources to complete the projects. The projects are implemented by the participants, but should involve the entire laboratory staff. Participants present their findings at national or regional workshops or on a day set aside by the laboratory (facility-based approach).

FIND found that often the choice of improvement projects does not reflect the priority gaps of the laboratory. In 2015, FIND adopted a more stringent criterion for improvement project selection. Under the guidance of the certified trainers, each participant completes two improvement projects between trainings; both are ‘complex’ and require extensive planning and data collection. The first project is based on the subjects covered in the trainings. For example, Training 1 (*Quality indicators* and *Facilities and safety*), Training 2 (*Equipment*, *Purchasing and inventory*, and *Quality assurance*) and Training 3 (*Documents and records*, *Client Management and Customer Service*, and *Specimen management*) (Table 2). The second project addresses the weaknesses identified during the baseline assessment. These non-conformities are split between the participants and a different section of the TB SLMTA Harmonized Checklist is covered between trainings.

TB SLMTA uses a short-term mentoring model instead of the embedded model encouraged by SLMTA. Mentoring visits are conducted by the trainers over two or three days. Each facility receives two visits between each workshop. The outcomes of the mentoring visits and, in particular, the progress with improvement projects, is monitored by the mentors for each laboratory, and any necessary support provided. Standardised data collection tools are used to record the findings of mentorship visits.

**TABLE 2:** Examples and types of improvement projects implemented in the TB SLMTA programme.

Type and period of implementation	Example
Training 1	
Quality indicators Facilities and safety	Conducting safety audits Implementing sample rejection quality indicator Implementing quality indicators for smear microscopy / Gene Xpert Implementing equipment downtime quality indicator Implementing quality indicator for culture turn-around time Implementing quality indicator for training in biosafety
Training 2	
Equipment Purchasing and inventory Quality assurance	Monitoring equipment maintenance Monitoring and documenting internal quality control Implementing competency assessments for tuberculosis diagnostic tests Monitoring the usage of microscopy stains
**Training 3**	
Documents and records Client management and customer service Specimen management	Improving tuberculosis culture and drug susceptibility testing referral system Conducting customer satisfaction survey Implementing internal audits Implementing a system for tracking referral results

TB SLMTA, Strengthening Tuberculosis Laboratory Management Toward Accreditation.

**FIGURE 2:**
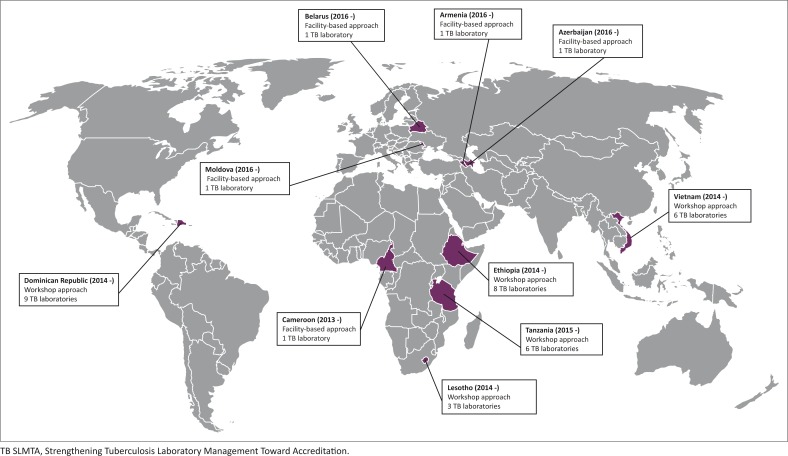
Implementation of TB SLMTA in 37 tuberculosis laboratories in 10 countries since 2013.

## Results from TB SLMTA implementation workshops

Since 2013, four regional TOTs have been conducted in Lesotho, Vietnam, South Africa and Moldova. Seventy trainees from 27 counties have been trained, and 59 are certified as trainers (including trainers under supervision), of which four participants are from WHO Supranational Reference Laboratories that provide tuberculosis laboratory technical support to countries. Twenty-six trainers are currently active in the TB SLMTA programme. Currently there are three Master Trainers. One Master Trainer, based in the African region, graduated after conducting a round of TB SLMTA, and we expect two more graduates in the coming year (one in the African region and one in South East Asia) for a total of six Master Trainers.

The TB SLMTA programme has been rolled out in 37 tuberculosis laboratories in 10 countries (Figure 2). National or regional TB SLMTA trainings using the workshop approach were conducted in 32 laboratories in five countries (Dominican Republic, Ethiopia, Lesotho, Tanzania and Vietnam). The facility-based approach has been used in one regional tuberculosis laboratory in Cameroon. The instructional phase is complete in these laboratories, but is ongoing in the four NTRLs in Eastern Europe (Armenia, Azerbaijan, Belarus and Moldova).

Baseline and exit assessment scores for 18 laboratories in four countries (Cameroon, Ethiopia, Lesotho and Tanzania) were available for analysis and are summarised in Table 3. At baseline, six of the eighteen laboratories had a zero-star rating, three had a one-star rating, seven had a two-star rating and two laboratories had a four-star rating. No laboratories had three- or five-star ratings at baseline assessment. At exit, two laboratories remained at zero stars, two were rated at one-star, four laboratories were rated at two stars, seven were rated at three-stars and three laboratories were rated at four-stars. The impact of TB SLMTA, as well as the individual country experiences will be addressed in separate publications.

FIND developed an online biosafety training programme in 2014,^24^ and TB SLMTA participants in Tanzania and Lesotho were enrolled in this training to complement the basic biosafety module of the TB SLMTA programme. This task-based online training was implemented in conjunction with biosafety improvements projects following Workshop 1.

Active participation for this extended time of the in-country training is a challenge for trainers and participants alike. In our cohort, 21 participants (Lesotho, 1; Dominican Republic, 8; Ethiopia, 7; Tanzania, 3; Vietnam, 2) were unable to complete the compulsory trainings and improvement projects due to personal or job-related reasons. Although in most cases, additional participants from the same laboratory meant that the laboratory was not excluded from continuing the programme, one regional tuberculosis laboratory in Tanzania was not able to complete the programme as both participants were unable to finish the training.

## Discussion

Tuberculosis laboratories are an essential element of tuberculosis prevention and care, providing testing for diagnosis, surveillance and treatment monitoring that can be accessible at all levels of the healthcare system. The TB SLMTA programme provides tuberculosis laboratories with customised support to accelerate the process of strengthening their QMS towards accreditation. There is an urgent need to expand the programme, as only 21 NTRLs (43%) on the African continent have received SLMTA training and only four NTRLs have reached accreditation. Although 44% of NTRLs report implementing a QMS, the extent of implementation is not known.^25^

**TABLE 3:** ‘Stars’ at baseline and exit for 18 tuberculosis laboratories in four countries completing the TB SLMTA programme (2013–2016).†

Number of stars	Baseline assessment	Exit assessment
0	6	2
1	3	2
2	7	4
3	0	7
4	2	3
5	0	0

TB SLMTA, Strengthening Tuberculosis Laboratory Management Toward Accreditation.

† , Results based on TB SLMTA Harmonized Checklist baseline and exit scores.

There were a number of challenges to implementing the TB SLMTA programme in the initial cohort of laboratories. The lack of experienced assessors was a challenge in some countries. SLIPTA-trained assessors with experience in tuberculosis testing were used to supplement certified TB SLMTA trainers. However, limited hands-on time spent with the TB SLMTA Harmonized Checklist during the TB SLMTA TOT, and SLIPTA trained assessors who are unfamiliar with implementing the tuberculosis laboratory specific clauses, may lead to inflated scoring during these assessments. While laboratories enrolled in the TB SLMTA programme may use the WHO Regional Office for Africa SLIPTA checklist, the additional components from GLI included in the TB SLMTA Harmonized Checklist v1.0 enable technical assessment alongside assessment of International Organization for Standardization components.

In instances where management had not been fully engaged in the TB SLMTA implementation, participants struggled to complete the improvement projects. It is therefore critical to actively engage upper management, both at the facility level and at the national Ministry of Health, to ensure their commitment to the programme. Institutionalisation of QMS into country programmes will be needed to support tuberculosis laboratories in achieving accreditation. Training and quality improvement activities may be seen as extra workload, especially in settings where staff shortage and high workload are existing challenges. Furthermore, trainers and mentors, who were critical components of the programme, are required to support the programme in addition to their usual duties. This may put additional strain on the laboratory as other staff are required to cover their workstations during their absence.

In addition to senior level engagement of the Ministry of Health, QMS activities being conducted by various implementing partners and donors should be coordinated centrally to ensure synergy to avoid duplication of effort and the risk of confusion and wastage of resources. We found multiple partners conducting overlapping activities related to QMS without clear coordination to ensure cost-efficiency and maximum impact from available resources. Partners should seek active collaboration on QMS activities, harmonisation of approaches and contributions of various groups, under the leadership and coordination of the Ministry of Health.

The TOTs are highly interactive, and some trainees whose first language is not English find the training challenging. Introduction of language proficiency and an introduction to QMS online training in 2014, helped ensure that trainees in the TOTs were successfully certified as trainers. However, this approach limits potential trainees. In 2016, FIND conducted a TOT in English, with real-time Russian translation (using a tuberculosis laboratory specialist as translator). All the trainees passed, suggesting that the model can be expanded to non-English speaking countries using translated materials (including the TB SLMTA Harmonized Checklist) and real-time translation. Careful considerations must be given to the translator, with preference given to those who have an insight into laboratory testing or QMS. Further analysis of this approach is required. Master Trainers are certified after successful supervision of the roll-out of the TB SLMTA programme in a country. To facilitate the expansion of the TB SLMTA programme, there is a need for more Master Trainers, particularly those that can train in languages other than English.

As noted earlier, FIND recently adopted a more stringent criterion for improvement project selection. A focus on the weaknesses identified in baseline assessment, in particular quality indicator and quality control monitoring and safety in the tuberculosis laboratory, has the potential to improve the impact of the TB SLMTA programme. As the cohort of tuberculosis laboratories that have used this strategy increases, the impact will be measured.

Mentoring of laboratories was found to be an important component to successful implementation of SLMTA. Embedded mentorship has proven to result in measurable improvement in the QMS in many countries, including Lesotho, Zimbabwe, Kenya and Nigeria.^26,27,28,29^ In TB SLMTA, certified trainers mentor participants during site visits and remotely between workshops. This short-term mentoring model is cost-effective, scalable and sustainable, and is well suited to the workshop approach of implementation used in our cohort. Ongoing structured mentoring of the tuberculosis laboratories that obtained four-star ratings at TB SLMTA exit assessment is being conducted in preparation for accreditation. The TB SLMTA programme is currently focused on tuberculosis laboratories with the capacity to perform advanced diagnostics such as culture and drug susceptibility testing. Tuberculosis laboratories on the lower level of the healthcare system may consider integration into current SLMTA activities. In addition, if feasible, countries should consider sharing mentoring and assessments between programmes. These cost-cutting approaches have an added benefit of integrating services and present opportunities for knowledge sharing and will encourage sustainability and institutionalisation of QMS.

### Limitations

This study is subject to a number of limitations. Firstly, none of the TB SLMTA laboratories have reached accreditation yet, and we are thus reporting on intermediate measures of quality improvement leading to the ultimate target of accreditation. Secondly, quality improvement from three stars to five stars (which is considered equivalent to accreditation readiness) is challenging.^30^ Thirdly, the role of mentorship in this final phase is still to be determined. Finally, in this article we have not addressed the costs of TB SLMTA. A cost estimation exercise is being undertaken. We do not expect the costs to differ substantially from costs of the SLMTA programme as reported by others.^31^

### Conclusions

TB SLMTA is a structured training and mentoring programme that is customised to meet the needs of tuberculosis laboratories in implementing a QMS in tuberculosis laboratories in resource-limited settings within a reasonably short time frame building a foundation toward further quality improvement toward achieving accreditation. Expansion of this programme is an urgent priority to address the need for accreditation of tuberculosis laboratories on the African continent and beyond.

BOX 1 Lessons Learned

- A structured training and mentoring programme can build a foundation toward further quality improve-ment in tuberculosis laboratories
- Structured mentoring, and institutionalisation of QMS into country programmes, is needed to support tu-berculosis laboratories to achieve accreditation.
